# Induced pluripotent stem cell-derived hepatocytes reveal TCA cycle disruption and the potential basis for triheptanoin treatment for malate dehydrogenase 2 deficiency

**DOI:** 10.1016/j.ymgmr.2024.101066

**Published:** 2024-02-23

**Authors:** Déborah Mathis, Jasmine Koch, Sophie Koller, Kay Sauter, Christa Flück, Anne-Christine Uldry, Patrick Forny, D. Sean Froese, Alexander Laemmle

**Affiliations:** a University Institute of Clinical Chemistry, Inselspital, Bern University Hospital, University of Bern, Switzerland; b Division of Pediatric Endocrinology, Diabetology and Metabolism, Department of Pediatrics, Inselspital, Bern University Hospital, University of Bern, Bern, Switzerland; c Pharmacy, Medical Faculty, University of Bern, Bern, Switzerland; d Proteomics and Mass Spectrometry Core Facility, Department for BioMedical Research (DBMR), University of Bern, Bern, Switzerland; e Division of Metabolism and Children's Research Center, University Children's Hospital, University of Zurich, Zurich, Switzerland; f Department of Cell Biology and Physiology, Washington University School of Medicine, St. Louis, MO, USA

**Keywords:** Malate aspartate shuttle, Malate dehydrogenase 2 deficiency, Human induced pluripotent stem cell technology, hiPSC-derived hepatocytes, Proteomics, Metabolic profiling, Triheptanoin

## Abstract

Mitochondrial malate dehydrogenase 2 (MDH2) is crucial to cellular energy generation through direct participation in the tricarboxylic acid (TCA) cycle and the malate aspartate shuttle (MAS). Inherited MDH2 deficiency is an ultra-rare metabolic disease caused by bi-allelic pathogenic variants in the *MDH2* gene, resulting in early-onset encephalopathy, psychomotor delay, muscular hypotonia and frequent seizures. Currently, there is no cure for this devastating disease. We recently reported symptomatic improvement of a three-year-old girl with MDH2 deficiency following treatment with the triglyceride triheptanoin. Here, we aimed to better characterize this disease and improve our understanding of the potential utility of triheptanoin treatment. Using fibroblasts derived from this patient, we generated induced pluripotent stem cells (hiPSCs) and differentiated them into hepatocytes (hiPSC-Heps). Characterization of patient-derived hiPSCs and hiPSC-Heps revealed significantly reduced MDH2 protein expression. Untargeted proteotyping of hiPSC-Heps revealed global dysregulation of mitochondrial proteins, including upregulation of TCA cycle and fatty acid oxidation enzymes. Metabolomic profiling confirmed TCA cycle and MAS dysregulation, and demonstrated normalization of malate, fumarate and aspartate following treatment with the triheptanoin components glycerol and heptanoate. Taken together, our results provide the first patient-derived hiPSC-Hep-based model of MDH2 deficiency, confirm altered TCA cycle function, and provide further evidence for the implementation of triheptanoin therapy for this ultra-rare disease.

**Synopsis:**

This study reveals altered expression of mitochondrial pathways including the tricarboxylic acid cycle and changes in metabolite profiles in malate dehydrogenase 2 deficiency and provides the molecular basis for triheptanoin treatment in this ultra-rare disease.

## 1 Introduction

**Fig. 1:**
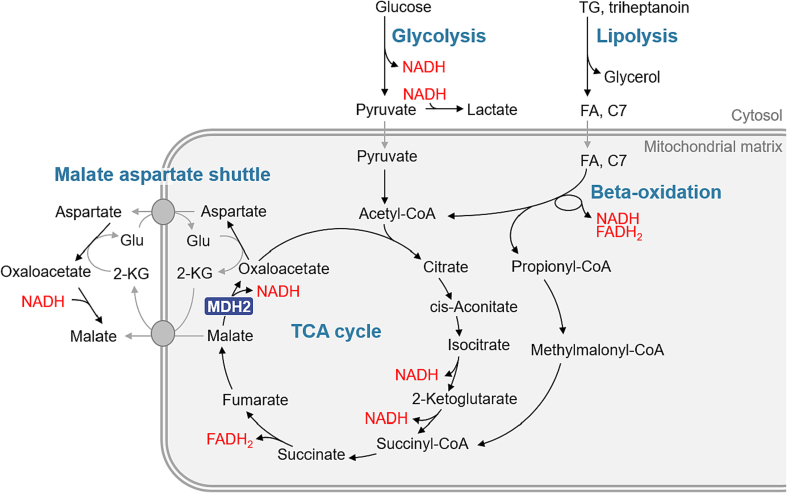
Scheme showing energy metabolism pathways, the MDH2 enzyme and the rational for triheptanoin treatment as alternative energy fuel. FA: fatty acid; C7: heptanoate; Glu: glutamate; 2-KG: 2-ketoglutarate.

Mitochondrial malate dehydrogenase 2 (MDH2) catalyzes the reversible oxidation of L-malate to oxaloacetate. This reaction is essential to cellular energy generation through direct participation in both the tricarboxylic acid (TCA) cycle and the malate aspartate shuttle (MAS) (Fig. 1) [1]. The MAS is constituted by the four enzymes MDH1, MDH2, GOT1 and GOT2 and two mitochondrial transporters. The oxoglutarate/malate carrier (OGC) is encoded by the gene *SLC25A11* and the two isoforms of the aspartate glutamate carrier (AGC1, also known as Aralar) and AGC2 (also known as Citrin) are encoded by the genes *SLC25A12* and *SLC25A13*, respectively. Their roles are to exchange reducing equivalents between the cytosol and the mitochondria, restoring NADH in mitochondria to provide electrons for oxidative phosphorylation and supporting regeneration of cytosolic NAD^+^ [2,3].

Inherited deficiency of MDH2 is an ultra-rare metabolic disease. Thus far 12 individuals have been described with bi-allelic pathogenic variants in the *MDH2* gene [[4], [5], [6], [7]]. MDH2 dysfunction causes an energy deficit affecting many organs, leading to symptoms including early-onset encephalopathy, frequent seizures, psychomotor delay, and muscular hypotonia [4], although symptom onset and affected organs may vary by individual [6,7]. To date, there is no curative treatment for this devastating disease. In MDH2 deficiency, oxidation of glucose and concomitant cytosolic generation of NADH, is impaired due to defective/inefficient shuttling of NADH reducing equivalents into mitochondria. Restoration of the energy deficit by alternative fuels to glucose has been proposed, and introduction of a high-fat, low carbohydrate (ketogenic) diet seems to be beneficial in MDH2 deficiency [4] and other disorders of the MAS [8]. Beta-oxidation of fatty acids generates FADH_2_ and NADH within the mitochondria, thereby circumventing the defective MAS in MDH2 deficiency (Fig. 1). Ketogenic diet has been applied in three individuals with MDH2 deficiency [4]. Although two individuals showed reduction in seizures, one of them died shortly after the introduction of the ketogenic diet due to metabolic decompensation.

In a recent report, our group described a patient affected with MDH2 deficiency who displayed improvement of clinical symptoms following treatment with the triglyceride triheptanoin [5]. Triheptanoin is composed of three seven‑carbon fatty (heptanoic) acids esterified to a glycerol backbone. Mitochondrial beta-oxidation of heptanoate generates acetyl-CoA and propionyl-CoA, which can subsequently enter the TCA cycle as anaplerotic substances (Fig. 1), while the glycerol may participate in the glycerol phosphate shuttle or be used for gluconeogenesis [[9], [10], [11]]. Together, these may help to maintain an appropriate energy balance.

In this work, we aimed to better characterize MDH2 deficiency and its amenability to treatment with triheptanoin. We generated induced pluripotent stem cells (hiPSCs) derived from fibroblasts of the MDH2-deficient patient and differentiated them into hepatocytes (hiPSC-Heps). Characterization of the generated hiPSC-Heps using western blot analysis, untargeted proteotyping and metabolic profiling indicated mitochondrial dysregulation, including TCA cycle and MAS abnormalities. Treatment with the components of triheptanoin (heptanoate and glycerol) normalized some of the metabolite disturbances observed in TCA cycle and MAS.

## 2 Material and methods

### 2.1 Patient

Written informed consent was obtained from the patient and approved by the local ethics committee in Bern, Switzerland (project ID: 2020–02979). Skin fibroblasts were gained by skin punch biopsy.

As previously reported [5], our currently 7-year-old female patient was diagnosed with MDH2 deficiency when she was 18 months old and suffered from metabolic decompensation resulting in a stroke-like episode. Molecular genetic testing using trio-whole exome sequencing revealed compound heterozygous missense variants in the *MDH2* gene: c.398C > T, p.(Pro133Leu) inherited from her mother and c.445delinsACA, p.(Pro149Thrfs*22) inherited from her father. According to ACMG guidelines both variants were classified as pathogenic. The patient is currently under treatment with triheptanoin oil (Ultragenyx pharmaceutical Inc., CA, USA) as previously described [5]. From a clinical perspective she is developing well and does not show any side effects related to this individual drug trial.

### 2.2 Generation and characterization of hiPSCs and differentiation into hiPSC-Heps

Fibroblasts from our MDH2 deficient patient were reprogrammed into hiPSCs (designated MDH2D hiPSCs) and cultured as previously described [[12], [13], [14]]. hiPSCs from a healthy 30-year-old Japanese male (Coriell Repository (#GM25256)) were used as control line (designated Ctrl hiPSCs). hiPSCs were subsequently differentiated into hiPSC-Heps recapitulating embryonic development stages according to our previously established and published protocol [12,14]. Pluripotency markers of hiPSCs and hepatocyte-specific markers of hiPSC-Heps were analyzed by quantitative real-time qPCR after isolating mRNA transcribed into cDNA as previously described [12].

### 2.3 Cell culture experiments and reagents

Heptanoate and glycerol (both chemicals from Sigma Aldrich) stock solutions were prepared in DMSO at stock concentrations of 200 mM for heptanoate and 60 mM for glycerol. Prior to use, stock solutions were diluted 1000-times in the appropriate cell culture medium to receive working concentrations of 200 μM for heptanoate and 60 μM for glycerol. After 21 days of hepatocyte differentiation, cells were treated with 200 μM heptanoate with or without addition of 60 μM of glycerol or with DMSO only (controls) for 24 h prior to cell harvest and cell lysate preparation. After removing cell culture supernatants, cells were washed 1× with ice-cold PBS and 40 to 70 μL of ice-cold RIPA lysis buffer added per well of a 6-well-plate and cells collected with a cell scraper. Cell lysates were collected in microfuge tubes, kept on ice for 15 min, vortexed every 5 min to disrupt cell membranes prior to centrifugation at 17`000 *g* for 15 min at 4 °C and transferred into new ice-cold microfuge tubes. Protein concentrations were determined according to the instructions of the DC Protein Assay Kit (Bio Rad). The reaction is based on the established Lowry method as previously described [15]. Aliquots were prepared for further analyses including western blotting, proteomics and metabolic profiling (see below for extraction prior to metabolic profiling).

### 2.4 Western blot

Protein expression was analyzed by western blotting using a 10% SDS-PAGE and transferred on a Immobilon®-P polyvinylidene fluoride membrane (Merck Millipore) using the Mini-PROTEAN system (BIO-RAD) in 1× turbo transfer buffer (25 mM TRIS, 192 mM glycine, 0.01% SDS, 20% methanol (Merck Millipore). Membranes were blocked in 5% milk in Tris buffered saline (TBS) containing 0.1% tween-20 (TBST) buffer for one hour at room temperature. Membranes were incubated with primary antibodies at 4 °C overnight and washed three times with 1xTBST. The same process was repeated with the secondary antibody. Details regarding antibodies can be found in the Suppl. Table 1. Signals were revealed using ECLTM Prime western blotting detection reagents (Amersham) accordingly to the manufacturer's instructions. Images were captured with a Fusion Solo 6S imager (Vilber Lourmat S.A.) and quantified using the open-source software ImageJ.

### 2.5 Untargeted proteotyping

Samples were diluted 2-times with 4% SDS/20 mM DTT, incubated at 75 °C for 10 min, alkylated with 1/10 volume of 0.5 M IAA/50 mM, Tris-HCl pH 8 at room temperature for 30 min and precipitated with 5 volumes acetone at −20 °C for 2 h. The pellets were then resuspended in 8 M urea/50 mM Tris pH 8 and an aliquot corresponding to 5 μg protein was digested by trypsin at room temperature overnight. The digests were analyzed by liquid chromatography (LC)-mass spectrometry (MS)/MS (Ultimate 3000 coupled to a QExactive HF mass spectrometer, ThermoFisher Scientific, Reinach, Switzerland) with three injections of 5 μL digests. Peptides were trapped on a μPrecolumn C18 PepMap100 (5 μm, 100 Å, 300 μm × 5 mm, ThermoFisher Scientific, Reinach, Switzerland) at a flow rate of 10 μL/min with 0.05% TFA in water/acetonitrile 98:2 (*v*/v) and separated by backflush on a C18 column (5 μm, 100 Å, 75 μm × 15 cm, C18, NIKKYO TECHNOS CO., LTD.) by applying a 90-min gradient of 5% acetonitrile to 40% in water, 0.1% formic acid, at a flow rate of 350 nL/min. The Full Scan method was set with resolution at 60`000 with an automatic gain control (AGC) target of 1E06 and maximum ion injection time of 50 ms. The data-dependent method for precursor ion fragmentation was applied with the following settings: resolution 15`000, AGC of 1E05, maximum ion time of 110 ms, mass window 1.6 *m*/*z*, collision energy 28, under fill ratio 1%, charge exclusion of unassigned and 1+ ions, and peptide match preferred, respectively; match between runs was enabled but constrained to group replicates. The samples were then searched and quantified with MaxQuant[maxquant] version 1.6.14.0, against the swissprot[uniprot] human database (including isoforms) release 2022_01, to which common contaminants were added [16,17]. The following search parameters were applied: carbamidomethylation on cysteine was set as a fixed modification, methionine oxidation and protein N-terminal acetylation as variable modifications; first search peptide and MS/MS match tolerance were set respectively to 10 and 20 ppm; digestion enzyme was trypsin, with a maximum of 3 missed cleavages; protein groups with <2 peptides were discarded. For further analysis, potential contaminants and protein groups only detected by site were ignored. MaxQuant's Label Free Quantification (LFQ values were imputed (iLFQ) in the following manner: if there was at most 1 non-zero value in the group or replicates, then the remaining missing values were imputed by drawing a random value from a Gaussian distribution of width 0.3 x sample standard deviation and centered at the sample distribution mean minus 2.5 x sample standard deviation [16]. Any remaining missing values are imputed by the Maximum Likelihood Estimation method.

### 2.6 Metabolic profiling

Metabolic profiling was performed using two fully quantitative LC-MS/MS panels. The first panel included TCA cycle metabolites and ketone bodies and the second panel amino acids. Oxaloacetate was not included in the TCA cycle metabolite panel due to its instability and alpha-ketoglutarate was not detected in any cell lysate due to its relatively high quantification limit. 20 μL of cell lysates with known protein concentrations were diluted with 100 μL of perchloric acid (PCA) 0.6 M solution. Samples were vortexed, kept on ice for 15 min, centrifuged at 4 °C and 15′000 g and supernatants transferred for further analysis. For the first panel, 50 μL of extracts were mixed with 50 μL internal standard solution in 0.6 M PCA and 2 μL were injected into the LC-MS system (Shimadzu Nexera X2 LCX30 coupled to AB Sciex 6500+ Qtrap). Metabolites were analyzed in negative ion mode as described previously [18,19]. Data was acquired with Analyst (version 1.7) and processed with SCIEX OS (version 3.1). For the amino acid panel, 20 μL of extract were diluted with 380 μL internal standard solution in acetonitrile, 20 μL of the resulting solution was further diluted with 80 μL acetonitrile and 2 μL were injected into the LC-MS system (Waters Acquity I-Class system coupled to Waters Xevo TQ-S). Amino acids were analyzed in positive ion mode as described previously [20]. Data were acquired with MassLynx (version 4.1, Waters) and processed with TargetLynx. Normalization of metabolite concentrations to protein concentrations enabled fully quantitative data.

### 2.7 Statistics

Statistical analyses were performed for all experiments containing at least three replicates using R v4.3.1 or GraphPad Prism and Student *t-*test. For multiple group comparisons, ordinary one-way ANOVA or Kruskal-Wallis tests were preferred and corrected with Dunnett's test. Data are expressed as mean ± standard error of mean (SEM). Threshold value of *p* < 0.05 was considered significant.

## 3 Results

### 3.1 Generation and characterization of hiPSCs and hiPSC-Heps

**Fig. 2:**
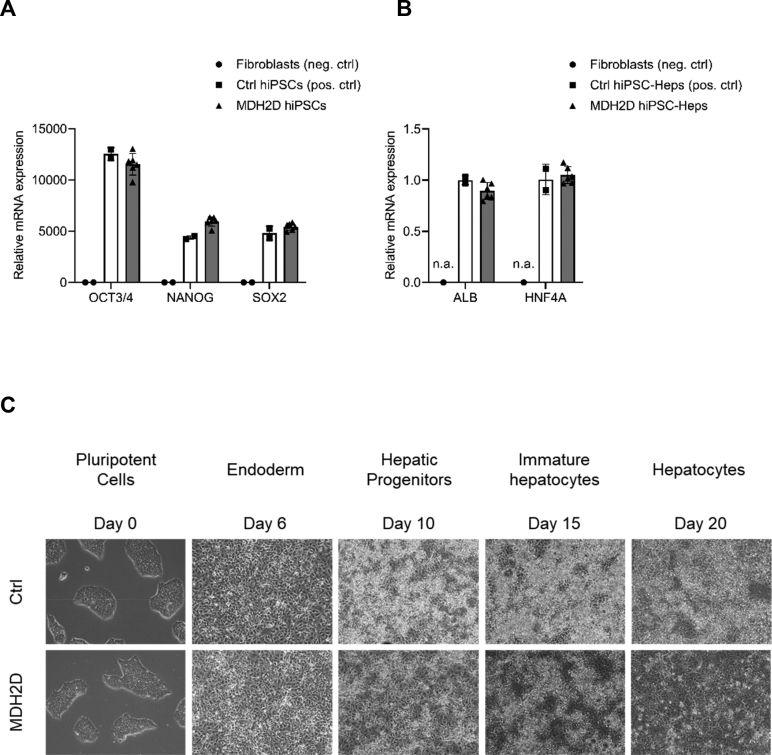
hiPSCs and hiPSC-Heps generation and characterization. (A) Relative mRNA expression levels of pluripotency markers OCT3/4, NANOG and SOX2 in MDH2D hiPSCs and in fibroblasts which were used as negative control (neg. ctrl) and in previously generated and characterized Ctrl hiPSCs which were used as positive control (pos. ctrl) for pluripotency marker expression. (B) Relative mRNA expression levels of hepatocyte-specific markers ALB and HNF4A in MDH2 hiPSC-Heps and the same control samples as described in (A). n.a.; no amplification. (C) Representative images of Ctrl (upper) and MDH2D (lower) hiPSCs through-out the differentiation process to hiPSC-Heps, illustrating cell morphological changes.

We previously described an 18-month-old girl with a stroke-like episode who was diagnosed with MDH2 deficiency and showed positive clinical response to triheptanoin treatment which was initiated at 36 months of age [5]. Applying episomal reprogramming [[12], [13], [14]] on patient-derived dermal fibroblasts from this patient, we generated hiPSCs (designated MDH2D hiPSCs) and compared them to a previously generated hiPSC line (designated Ctrl hiPSCs). As expected, both MDH2D and Ctrl hiPSCs showed endogenous expression of pluripotency markers (OCT3/4, NANOG and SOX2) not identified in fibroblasts (Fig. 2A). Following our previously established protocol [12,14], we differentiated both patient and control hiPSCs towards a hepatocyte lineage (hiPSC-Heps). hiPSC-Heps revealed endogenous expression of markers typical of hepatocytes (ALB and HNF4A) (Fig. 2B). Both hiPSCs and hiPSC-Heps showed morphological appearance characteristic of their cell types (Fig. 2C).

### 3.2 Patient-derived hiPSC-Heps have reduced MDH2 protein

**Fig. 3:**
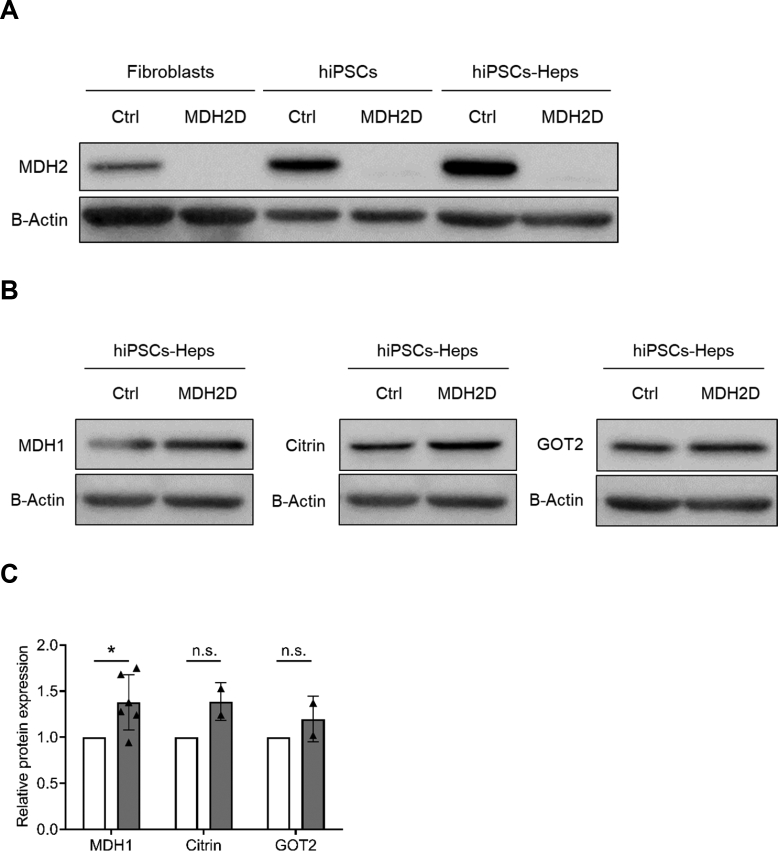
Western blot analysis of MDH2 and additional components of the malate aspartate shuttle. (A) MDH2 protein levels in MDH2D fibroblasts, induced pluripotent stem cells (hiPSCs) and differentiated hepatocytes (hiPSC-Heps) along with corresponding Ctrl, as determined by western blot. Two further replicates are presented in Supp. Fig. 1A. (B, C) MDH1, Citrin and GOT2 protein levels in MDH2D and Ctrl hiPSC-Heps as determined (B) and quantified (C) by western blot. A further replicate as well as western blot of each protein in fibroblasts and hiPSCs are presented in Supp. Fig. 1B. For both (A) and (B), B-Actin was used as loading control/housekeeping protein. For (B), MDH1 and Citrin was analyzed on the same membrane, therefore an identical image for B-Actin is shown here.

The affected individual was compound heterozygous for the *MDH2* variants c.398C *>* T, p.(Pro133Leu) and c.445delinsACA, p.(Pro149Thrfs*22) [5]. To determine the effect of these changes at the protein level, and to verify that these pathogenic variants were conserved in hiPSCs and hiPSC-Heps, we performed western blotting analysis using an antibody targeted to human MDH2. Compared to Ctrl, we found apparently reduced MDH2 protein levels in MDH2D fibroblasts, hiPSCs and hiPSC-Heps (Fig. 3A and Suppl. Fig. 1A). As a first assessment of whether there might be compensatory changes in associated proteins, we examined protein levels of the MAS proteins MDH1, a cytosolic isoenzyme of MDH2, as well as Citrin (AGC2) and GOT2. While western blotting analysis revealed a potential increase in MDH1 and Citrin protein expression, GOT2 protein expression did not appear to be altered when comparing MDH2D and Ctrl hiPSC-Heps (Fig. 3B and C, and Suppl. Fig. 1B).

### 3.3 Untargeted proteotyping reveals mitochondrial and TCA cycle dysregulation

**Fig. 4:**
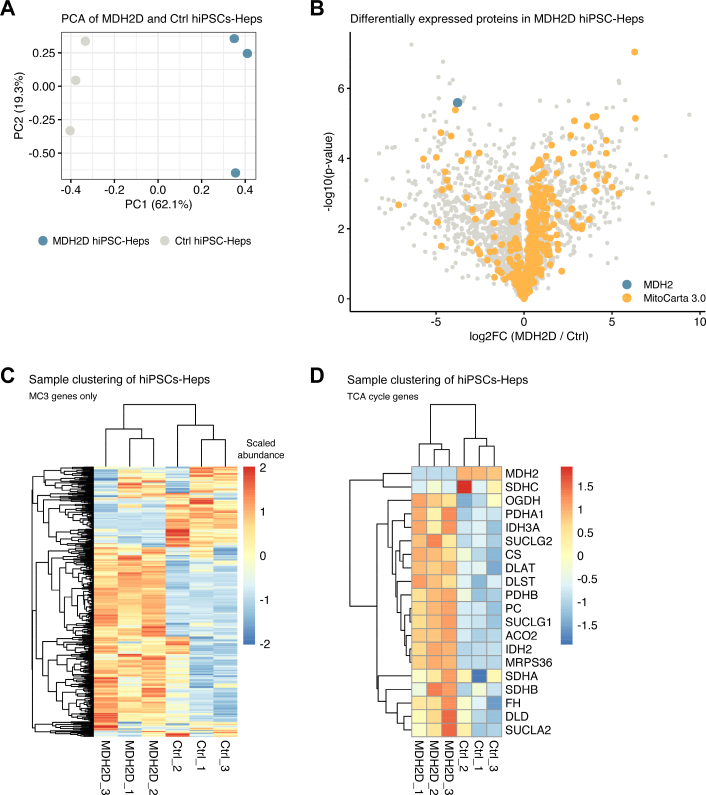
Untargeted proteotyping reveals altered mitochondrial protein levels in MDH2-deficient patient-derived hiPSC-Heps. (A) Principal component analysis (PCA) revealing clear separation of MDH2D and Ctrl hiPSC-Heps. (B) Volcano plot demonstrating differential protein expression in MDH2D and Ctrl hiPSC-Heps. Mitochondrial proteins, as defined by MitoCarta3.0 are in orange, MDH2 in teal. Two-sided *t*-test was used to calculate *p*-values. (C and D) Sample clustering of mitochondrial (C) and specifically TCA cycle enzymes (D) in MDH2D and Ctrl hiPSC-Heps. Three replicates per cell line are represented.

To further examine protein differences in MDH2D hiPSC-Heps, we used untargeted proteotyping. Overall, we captured protein levels of 2974 genes in MDH2D and Ctrl samples (Suppl. Table 2). Principal component analysis revealed clear separation of MDH2D and Ctrl samples (Fig. 4A). This global difference between samples was further supported by unguided hierarchical clustering (Suppl. Fig. 2A). Examination of differentially expressed proteins revealed an increased abundance of mitochondrial proteins, as defined by MitoCarta3.0 [21], in MDH2D compared to Ctrl hiPSC-Heps (Fig. 4B and C). Within this subset were proteins involved in fatty acid oxidation (Suppl. Fig. 2B) and oxidative phosphorylation (Suppl. Fig. 2C). While MDH2 levels were decreased as expected, nearly all other TCA cycle enzymes were increased (Fig. 4D).

### 3.4 Metabolite profiling confirms TCA cycle dysregulation, which is ameliorated by treatment with triheptanoin components heptanoate and glycerol

**Fig. 5:**
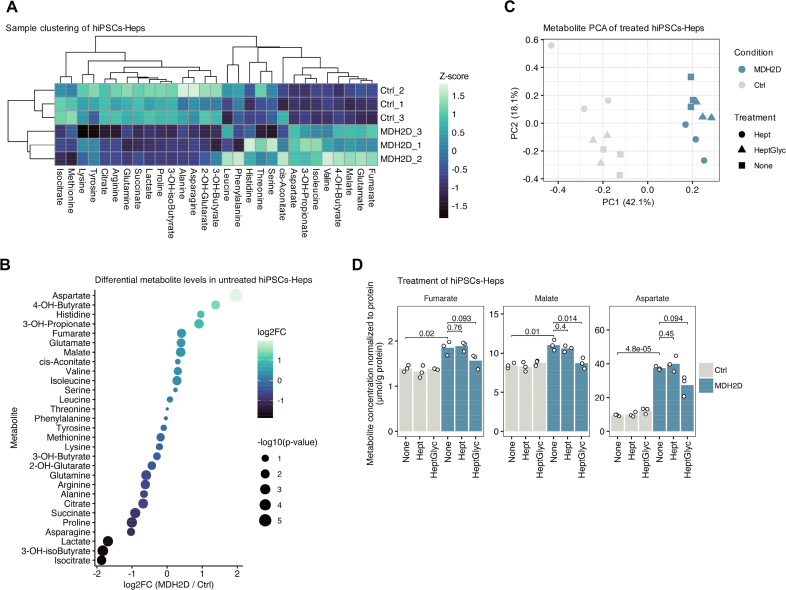
Untargeted metabolite profiling of MDH2-deficient patient-derived hiPSC-Heps. (A) Sample clustering of metabolites determined in MDH2D and Ctrl hiPSC-Heps. (B) Differential metabolite concentrations in untreated MDH2D and Ctrl hiPSC-Heps. (C) Principal component analysis (PCA) of metabolite concentrations showing separation of MDH2D and Ctrl hiPSC-Heps upon treatments. Treatments were either heptanoate alone (Hept) or heptanoate and glycerol combined (HeptGlyc) or solvent only (=DMSO; none). (D) Response of fumarate, malate and aspartate to treatments. Other measured metabolites are provided in Suppl. Fig. 3. Three replicates per cell line are represented.

To examine the functional consequence of MDH2 deficiency, we performed intracellular metabolite profiling using targeted LC-MS/MS methods ([[18], [19], [20]]/see Methods). MDH2 is an integral enzyme of the TCA cycle and is directly involved in production of NADH (Fig. 1). Based on this rationale, as well as our observation of elevated expression of TCA cycle proteins, we focused our metabolite panel on molecules that are related to the TCA cycle (Fig. 5A). Global analysis through unguided hierarchical clustering identified clear separation between MDH2D and Ctrl hiPSC-Heps (Fig. 5A). Among the most dysregulated metabolites were aspartate, glutamate and malate, that are involved in the MAS, as well as fumarate, citrate and succinate, intermediates of the TCA cycle (Fig. 5B).

Since treatment with triheptanoin (composed of glycerol and three seven‑carbon fatty acids (heptanoate)) resulted in improvement of clinical symptoms in our patient, we examined the effect of these components in our in vitro model. Since we expected that hiPSC-Heps do not express the lipase required to enzymatically cleave triheptanoin, we directly provided the components glycerol and heptanoate. Application of glycerol and heptanoate (mimicking triheptanoin), or heptanoate only as control, resulted in essentially very minor changes to the overall metabolic profile as revealed by principal component analysis (Fig. 5C). There was no significant effect on the majority of metabolites measured in both MDH2D and Ctrl hiPSC-Heps (Suppl. Table 3 and Suppl. Fig. 3), apart from glutamine that was increased upon treatment in MDH2D but not in Ctrl. Furthermore, we found that heptanoate and glycerol, tend to reduce fumarate and aspartate and significantly reduced malate back towards Ctrl levels (Fig. 5D).

Taken together, treatment with glycerol and heptanoate revealed significant effects on specific metabolites, and trended to impact on aspartate, which is linked to the MAS directly, or the two TCA cycle metabolites fumarate and malate, which are linked to MDH2 enzyme.

## 4 Discussion

In the present study, we aimed to objectify our successful triheptanoin treatment trial performed in vivo in the MDH2 deficient patient [5]. By creating an in vitro liver model with patient-derived hepatocytes, we sought to delineate potential pathophysiological mechanisms involved in disease and to evaluate treatment response. Therefore, we generated fibroblast-derived hiPSCs from our MDH2 deficient patient which were differentiated into MDH2D hiPSC-Heps and treated with the components of triheptanoin – glycerol and heptanoate.

We previously established other liver disease models recapitulating the hepatic phenotype of specific rare inherited metabolic diseases, such as urea cycle disorders [12]. It is known from our studies as well as those performed by others [12,22] that hiPSC-Heps are not fully mature cells and compared to primary human hepatocytes retain fetal-like characteristics. Thus, potential limitations arise, e.g. specific disease-relevant genes might not be expressed. Here, we confirmed expression of MDH2 and other MAS proteins by western blotting. Next, by untargeted proteomics, we found that a vast majority of mitochondrial proteins were upregulated in MDH2D hiPSC-Heps. This might be the consequence of cells trying to bypass the defective MDH2 enzyme by upregulating alternative mitochondrial pathways for energy generation.

It was long suggested that TCA cycle defects are not compatible with life. In recent years, several TCA cycle defects (i.e. bi-allelic pathogenic variants in TCA cycle genes) have been discovered [[23], [24], [25], [26], [27], [28], [29], [30], [31], [32], [33], [34], [35], [36]]. A complete disruption of the TCA cycle would potentially result in a block of NADH generation and a lack of ATP production with deleterious consequences. Thus it seems likely that affected cells must upregulate alternative pathways in order to adapt and survive. Untargeted analysis of proteomics data from MDH2 hiPSC-Heps revealed upregulation of the fatty acid oxidation pathway compared to control cells. Since MDH2 deficiency causes a block in the TCA cycle, glucose oxidation as well as oxidation of several amino acids are potentially hampered. As mentioned above, beta-oxidation mediated breakdown of fatty acids – such as heptanoate – allows generation of FADH_2_ and NADH within the mitochondria, displaying one potential way in which MDH2 deficient cells could circumvent the enzymatic TCA cycle block. Further studies are required to assess metabolic flux in MDH2 deficient cells.

To further elucidate our MDH2 liver model, we analyzed several TCA cycle metabolites as well as amino and organic acids crucial to intermediary metabolism. Among the increased metabolites found in MDH2 deficient cells, were aspartate (consistent with previously reported results from other MAS defects, i.e. MDH1 deficiency [37]), glutamate, malate and fumarate; whereas succinate and citrate were decreased. Thus, the malate/succinate as well as the fumarate/succinate ratios were strikingly elevated in MDH2D hiPSC-Heps compared to controls, suggesting alternative usage of succinate. In line with this finding, we previously reported a decreased pyruvate/succinate respiration ratio in patient-derived fibroblasts [5]. Cells deficient in one of the TCA cycle enzymes, such as MDH2, tend to limit flux of pyruvate to complex I of the electron transport chain – i.e. reduce complex I-dependent respiration and energy generation. As a compensatory mechanism MDH2-deficient cells may prefer succinate as a substrate which is directly fed into complex II of the electron transport chain, avoiding/circumventing flux through the TCA cycle.

We observed a significant increase in glutamine upon treatment with heptanoate and glycerol in MDH2 deficient cells, whereas in healthy control hiPSC-Heps glutamine concentration did not alter in response to any of the applied treatments. One use of glutamine by cells is to employ glutaminolysis to serve as an anaplerotic source of TCA cycle substrates. Such an adaptive mechanism might be used in MDH2 deficiency to cope the lack of energy supply through other substrates. Further investigation may uncover whether the diminished consumption of glutamine in response to treatment, which was only observed in MDH2D hiPSC-Heps and not in control cells, is related to such a mechanism.

Normalization of aspartate, malate and fumarate was observed with addition of triheptanoin but not with heptanoate alone. A speculative mechanism underlying this could be that glycerol enters the glycerol phosphate shuttle restoring NAD^+^ in the cytosol and providing NADH for energy production in the mitochondria [38]. Furthermore, glycerol can also be used for pyruvate production [39]. In the mitochondria, pyruvate may be used for anaplerosis via pyruvate carboxylase to yield oxaloacetate entering the TCA cycle and bypassing the deficient MDH2 enzyme.

This study had a few important limitations. The main limitation is the availability of a single patient and control cell line. One further potential limitation of this work is the lack of age- and gender-matched controls. Furthermore from a clinical point of view it would be useful to investigate other cell types of affected organs such as neurons.

To conclude, in the present work we successfully generated an MDH2D liver disease model recapitulating disease-associated features such as reduced MDH2 expression and impaired flux through the MAS and the TCA cycle. Our established model allows us to study in greater detail the effects of triheptanoin as well as to test potential other dietary regimens. Currently performed and on-going experiments in our laboratory should shed light into new treatment strategies to treat this devastating disease.

## Funding

DSF is supported by the 10.13039/501100001711Swiss National Science Foundation (310030_192505) and the University Research Priority Program ITINERARE – Innovative Therapies in Rare Diseases. PF is supported by the 10.13039/100004410European Molecular Biology Organization (ALTF 263–2022) and the 10.13039/501100001711Swiss National Science Foundation (P500PB_211038). AL is supported by the Palatin Foundation (0087/2023).

## Informed consent and ethical approval

Written informed consent was obtained from the patient. This study was approved by the local ethics committee in Bern, Switzerland (project ID: 2020–02979).

## CRediT authorship contribution statement

**Déborah Mathis:** Writing – review & editing, Writing – original draft, Visualization, Validation, Project administration, Methodology, Investigation, Formal analysis, Data curation. **Jasmine Koch:** Formal analysis, Data curation. **Sophie Koller:** Formal analysis, Data curation. **Kay Sauter:** Supervision, Methodology, Conceptualization. **Christa Flück:** Writing – review & editing. **Anne-Christine Uldry:** Validation, Software, Methodology, Formal analysis. **Patrick Forny:** Writing – review & editing, Visualization, Software, Methodology, Funding acquisition, Formal analysis, Data curation. **D. Sean Froese:** Writing – review & editing, Writing – original draft, Funding acquisition, Formal analysis. **Alexander Laemmle:** Writing – review & editing, Writing – original draft, Visualization, Validation, Supervision, Resources, Project administration, Methodology, Investigation, Funding acquisition, Formal analysis, Data curation, Conceptualization.

## Declaration of competing interest

All authors declare no conflicts of interest.

## Data Availability

Data will be made available on request.

## Appendix A Supplementary data

Supplementary material Image 1
