# Supplementary material for: Induced pluripotent stem cell-derived hepatocytes reveal TCA cycle disruption and the potential basis for triheptanoin treatment for malate dehydrogenase 2 deficiency
Source: Mol Genet Metab Rep. 2024 Feb 23;39:101066. doi: 10.1016/j.ymgmr.2024.101066 (PMC10900122; doi:10.1016/j.ymgmr.2024.101066)
Supplement: Supplementary file 1 — Supplementary material [file mmc1.docx]

## **Supplementary Information**

## **Induced pluripotent stem cell-derived hepatocytes reveal TCA cycle disruption and the potential basis for triheptanoin treatment for malate dehydrogenase 2 deficiency**

Déborah Mathis, Jasmine Koch, Sophie Koller, Kay Sauter, Christa Flück, Anne-Christine Uldry, Patrick Forny, D. Sean Froese, Alexander Laemmle

**Contact Information**

**Corresponding author:** Alexander Laemmle

**Postal Address:** Department of Pediatrics; Julie-von-Jenner-Haus, Freiburgstrasse 15, 3010 Bern, Switzerland

**Phone:** +41 31 632 95 44

**E-mail:** alexander.laemmle@insel.ch

**Supplementary Tables**

| **Antibody target** | **Species / Produced in** | **Reference** | **Dilution** | **Supplier / Manufacturer** |
| --- | --- | --- | --- | --- |
| **MDH2** | Rabbit | ab181873 | 1:1’000 | Abcam |
| **MDH1** | Rabbit | ab180152 | 1:1’000 | Abcam |
| **GOT2** | Mouse | MA5-15595 | 1:1’000 | ThermoFisher |
| **Citrin** | Rabbit | ab167166 | 1:1’000 | Abcam |
| **Beta-Actin** | Mouse | mAbcam 8224 | 1:5’000 | Abcam |
| **Rabbit IgG** | Donkey | NA934V / (17550629) | 1:10’000 | Cytiva |
| **Mouse IgG** | Sheep | NA9310V / (17076040) | 1:10’000 | Cytiva |

**Supplementary Table 1. List of antibodies used in this study**

**Supplementary Table 2. Protein levels of all 2974 genes captured by untargeted proteotyping**

See separate file: Supplementary Table 2.csv

**Supplementary Table 3. Metabolite profiling of treated cells**

See separate file: Supplementary Table 3.csv

**Supplementary Figures**

**A**

**
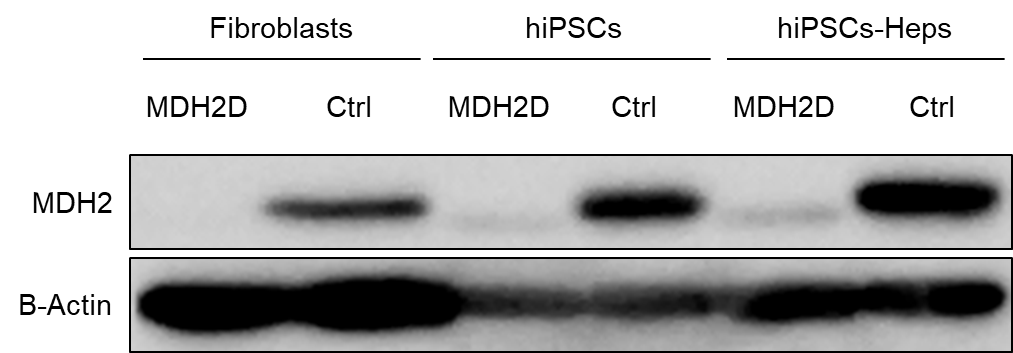
**

**
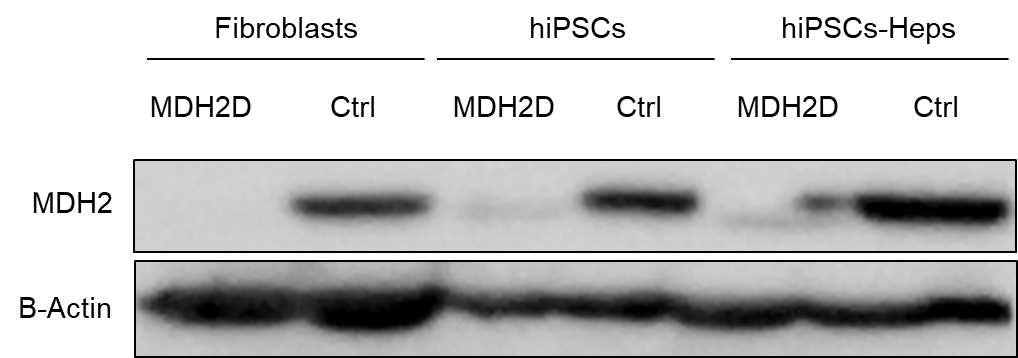
**

**B**


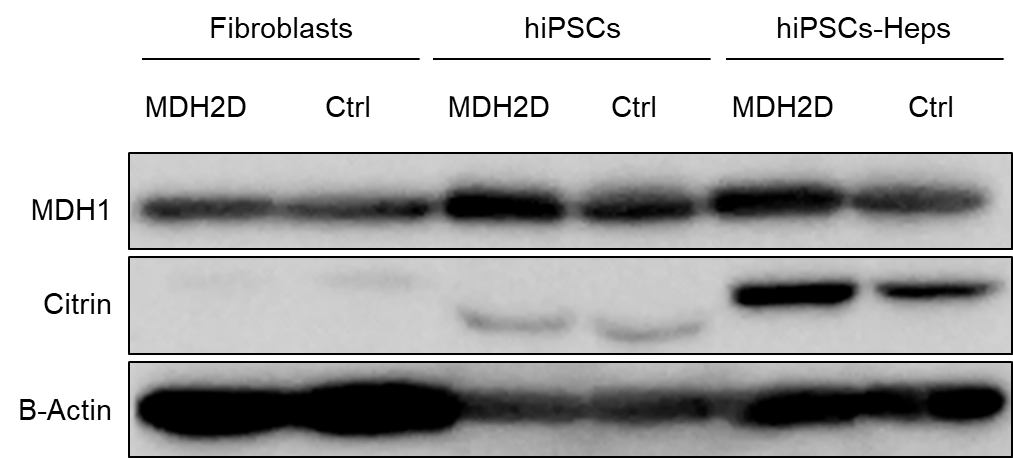


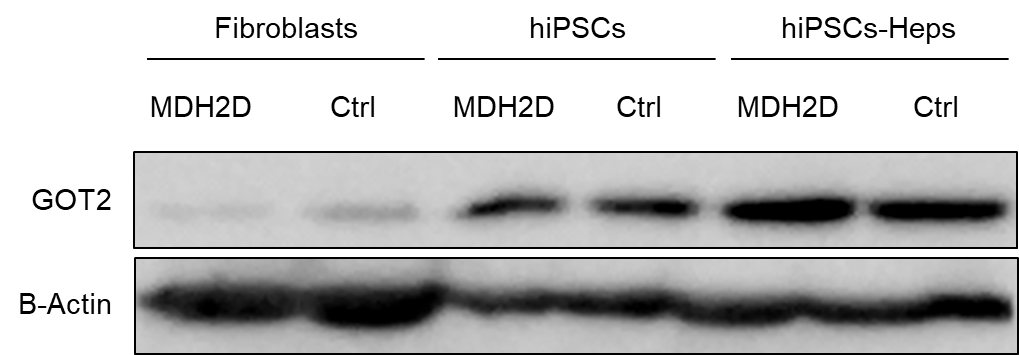


**Supplementary Figure 1. MDH2 and additional components of the malate aspartate shuttle**

(A) MDH2 protein levels in MDH2D fibroblasts, induced pluripotent stem cells (hiPSCs) and differentiated hepatocytes (hiPSC-Heps) along with corresponding Ctrl, as determined by western blot. Images here represent replicates two and three, and correspond to Figure 2A (total, n = three replicates). (B) MDH1, Citrin and GOT2 protein levels in MDH2D and Ctrl fibroblasts, hiPSCs and hiPSC-Heps as determined by western blot. Images here for hiPSC-Heps represent the second replicate and correspond to Figure 2B (total, n = two replicates).


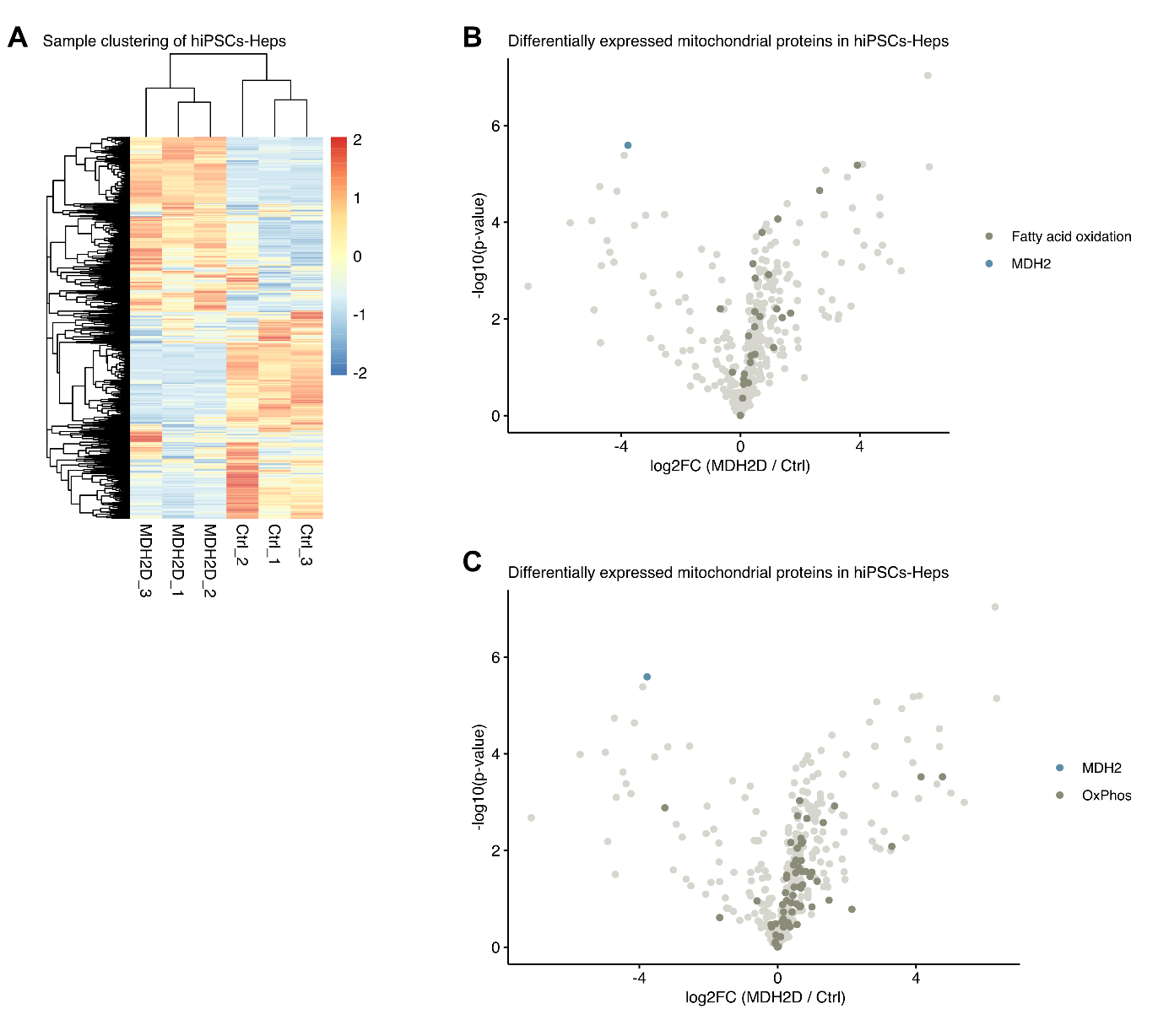


**Supplementary Figure 2. Proteomics sample clustering and targeted investigation of mitochondrial protein subsets**

(A) Hierarchical clustering using Euclidean distance of the entire proteomics dataset reveals global difference of protein expression between MDH2D and Ctrl hiPSC-Heps. (B and C) Volcano plots illustrating only the mitochondrial subset of proteins (see Figure 3B), for which fatty acid oxidation (B) and oxidative phosphorylation (C) proteins are emphasized as dark grey dots.





**Supplementary Figure 3. Effect of treatment on selected metabolite concentrations**

Effect of treatments, either heptanoate alone (Hept) or heptanoate and glycerol combined (HeptGlyc) or solvent only (=DMSO; none) on metabolite concentrations in both MDH2D and Ctrl hiPSC-Heps.
